# Nationwide in‐hospital mortality following colonic cancer resection according to hospital volume in Germany

**DOI:** 10.1002/bjs5.50173

**Published:** 2019-05-03

**Authors:** J. Diers, J. Wagner, P. Baum, S. Lichthardt, C. Kastner, N. Matthes, S. Löb, H. Matthes, C.‐T. Germer, A. Wiegering

**Affiliations:** ^1^ Department of General, Visceral, Vascular and Paediatric Surgery University Hospital, University of Würzburg Würzburg Germany; ^2^ Comprehensive Cancer Centre Mainfranken University of Würzburg Medical Centre Würzburg Germany; ^3^ Department of Biochemistry and Molecular Biology University of Würzburg Würzburg Germany; ^4^ Havelhöhe Community Hospital Berlin Germany

## Abstract

**Background:**

Colonic cancer is the most common cancer of the gastrointestinal tract. The aim of this study was to determine mortality rates following colonic cancer resection and the effect of hospital caseload on in‐hospital mortality in Germany.

**Methods:**

Patients admitted with a diagnosis of colonic cancer undergoing colonic resection from 2012 to 2015 were identified from a nationwide registry using procedure codes. The outcome measure was in‐hospital mortality. Hospitals were ranked according to their caseload for colonic cancer resection, and patients were categorized into five subgroups on the basis of hospital volume.

**Results:**

Some 129 196 colonic cancer resections were reviewed. The overall in‐house mortality rate was 5·8 per cent, ranging from 6·9 per cent (1775 of 25 657 patients) in very low‐volume hospitals to 4·8 per cent (1239 of 25 825) in very high‐volume centres (*P* < 0·001). In multivariable logistic regression analysis the risk‐adjusted odds ratio for in‐house mortality was 0·75 (95 per cent c.i. 0·66 to 0·84) in very high‐volume hospitals performing a mean of 85·0 interventions per year, compared with that in very low‐volume hospitals performing a mean of only 12·7 interventions annually, after adjustment for sex, age, co‐morbidity, emergency procedures, prolonged mechanical ventilation and transfusion.

**Conclusion:**

In Germany, patients undergoing colonic cancer resections in high‐volume hospitals had with improved outcomes compared with patients treated in low‐volume hospitals.

## Abstract (es)

**Antecedentes:**

El cáncer de colon es el cáncer más frecuente del tracto digestivo. El objetivo de este estudio fue determinar las tasas de mortalidad tras resección de cáncer de colon y el efecto del volumen de casos del hospital sobre la mortalidad intrahospitalaria en Alemania.

**Métodos:**

Los pacientes ingresados con el diagnóstico de cáncer de colon sometidos a resección colónica entre 2012 y 2015 se identificaron a partir de un registro nacional utilizando los códigos de los procedimientos. La medida de resultado fue la mortalidad intrahospitalaria. Los hospitales se clasificaron de acuerdo con su número de casos de resecciones de cáncer de colon y los pacientes fueron categorizados en 5 diferentes subgrupos en la base del volumen del hospital.

**Resultados:**

Se revisaron 129.196 resecciones de cáncer de colon. La tasa de mortalidad fue de 5,75%, variando desde 6,92% (*n* = 1.775) en hospitales de bajo volumen hasta 4,80% (*n* = 1.239) en centros con alto volumen, con una diferencia significativa entre los escenarios de bajo y alto volumen (*P* < 0,001). El análisis de regresión logística multivariable puso de manifiesto que la razón de oportunidades (*odds ratio*, OR) ajustada al riesgo de la mortalidad intrahospitalaria fue de 0,75 (i.c. del 95% 0,66‐0,84) en hospitales con volumen muy alto que realizaban más de 85,0 intervenciones/año, en comparación con hospitales de volumen muy bajo que realizaban menos de 13 intervenciones/año, tras ajustar por sexo, edad, comorbilidad, procedimiento urgente, ventilación mecánica prolongada y transfusiones.

**Conclusión:**

En Alemania, los pacientes sometidos a resección de cáncer de colon en hospitales de alto volumen tienen mejores resultados en comparación con los pacientes tratados en centros de bajo volumen.

## Graphical abstract

In Germany, perioperative mortality for colonic cancer resection at the national level is high. Patients undergoing resection of colonic cancer in high‐volume hospitals have improved outcomes compared with those treated in low‐volume hospitals.

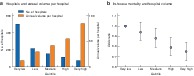

Mortality rate, hospital volume and CRC resections

## Introduction

Colorectal cancer is the most common malignancy of the gastrointestinal tract, affecting more than one million patients per year worldwide and accounting for more than 500 000 deaths^1^. Over the past two decades the introduction of membrane anatomy surgery with respect to embryological planes has led to improved long‐term survival rates but also increased perioperative morbidity and mortality^2^, ^3^, ^4^, ^5^, ^6^, ^7^. Although large multicentre studies have demonstrated low complication rates and a low 30‐day mortality rate after colorectal cancer resection^8^, these results could be biased by hospital volume. In addition, population‐based analyses suggest mortality rates of up to 6 per cent^9^, ^10^, ^11^, leading to an increased interest in quality assurance indicators in medicine and surgery.

A Cochrane review^12^ including nearly one million patients suggested that surgeon experience and hospital volume have a significant impact on short‐ and long‐term survival after colonic cancer surgery. Even though the German Cancer Society currently certifies centres with a minimum caseload of 50 colorectal cancer resections per year (30 colonic and 20 rectal), no official guidelines have been issued regarding the minimum number of patients with colonic cancer that should be treated annually per hospital, and around 50 per cent of the patients are still treated in non‐board‐certified hospitals.

The primary aim of this study was to analyse in‐house mortality following colonic cancer surgery in Germany according to hospital volume.

## Methods

Data of individual inpatients treated from January 2012 to December 2015 were obtained from the nationwide German diagnosis‐related group (DRG) statistics^13^. Inclusion criteria were a DRG code for colonic cancer (C18 as main diagnosis) and a colonic resection performed in a German hospital.

Procedure codes for colonic resection ranged from colectomy to resection of a colonic segment, with the exclusion of appendicectomy. Procedures were considered hierarchically, whereas more extensive resections were defined as the principal intervention to avoid double‐counting.

DRG data were accessed by controlled remote data analysis via the Research Data Centre of the Federal Statistical Office, in accordance with German legal data protection regulations. Data included secondary diagnoses, sex, patient age and duration of hospital stay. For case identification and data analysis, the German adaptation of ICD‐10 (ICD‐10‐GM) codes and German procedure codes (OPS codes) were used (versions 2012–2015; *Table* S1, supporting information)^14^. Analysis was restricted to patients with complete data records. When there were duplicate data, one data set was chosen at random and included for further analysis.

Hospitals were ranked according to their caseload for colonic cancer resections (continuous variable) and patients were categorized into five subgroups on the basis of hospital volume.

### Outcome measure

The main outcome of this study was in‐hospital mortality, defined as death while an inpatient irrespective of the actual length of hospital stay (LOS).

### Co‐morbidities

To account for differences in the co‐morbidity profile of patients between hospital volume quintiles, the co‐morbidity score was determined for each patient as proposed by Stausberg and Hagn^15^, based on ICD‐10 groups. Data on other potential confounders, such as sex, age or emergency procedures, were considered similarly and included in the analysis.

### Statistical analysis

In a first step, raw data were screened for missing values and checked for plausibility. The continuous variable age was recoded as a categorical dummy variable with three age categories (54 years or less, 55–74 years and 75 years or more). The cut‐offs were chosen based on pre‐existing epidemiological data, thereby assuring similar group sizes for the second and third age groups and confining patients with a presumably higher incidence of genetic aberration leading to early‐onset colonic cancer to one age group.

Patient characteristics were analysed descriptively for each year and according to hospital volume quintiles. Temporal trends and trends across volume categories were accessed by means of a non‐parametric test for trend, described elsewhere^16^.

Second, univariable odds ratios (ORs) between the main dependent variable (in‐house mortality) and the main independent variable (hospital volume quintile) were determined using Pearson's χ^2^ test or univariable logistic regression analysis, as appropriate. In addition, crude ORs between the secondary independent variables (listed below), the main independent variable and the outcome of interest were calculated to identify potential confounders. The possibility of important effect modification was assessed using the Mantel–Haenszel method, adjusting for each potential confounder. Correlation between each pair of variables was determined to detect multicollinearity.

The effect of hospital volume on in‐house mortality was evaluated by using a multivariable logistic regression model that included hospital volume as a random effect to account for clustering of patients in different institutions. The multivariable model was adjusted for known confounding effects such as sex, age, emergency procedures, co‐morbidity, mechanical ventilation for 48 h or more, and blood transfusion of 6 units or more. The model was also fitted with the number of patients per hospital as a continuous variable and with hospital volume quintile as a linear variable. Likelihood ratio tests were used to assess the model's fit and to evaluate the presence of linear trends.

The accuracy of the random‐effects estimators of the multivariable regression models was checked by refitting the models for different numbers of points and subsequent comparison of the values of the estimators. A maximum relative difference of 10^−4^ or less between the different quadrature points was considered acceptable.

Where appropriate, 95 per cent confidence intervals and *P* values were calculated. *P* < 0·050 was considered significant. All calculations were conducted using Stata® version 14.2 (StataCorp, College Station, Texas, USA).

## Results

**Figure 1:**
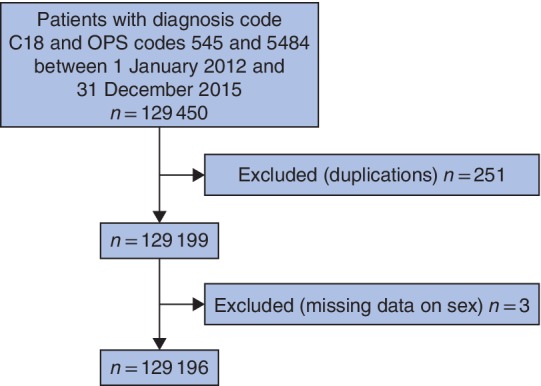
Flow diagram of patient selection

A total of 129 450 patients with a diagnosis of colonic cancer (ICD C18) who had colonic resection (relevant subgroups of OPS codes 545 and 5484) between 1 January 2012 and 31 December 2015 were identified from the nationwide DRG database of the German Federal Statistical Office. Missing data or duplicates occurred in 0·2 per cent (254 patients), leaving 129 196 patients for further analysis (*Fig*. 1).

The mean annual number of cases/hospital was 30·1 (*Table* 1). Some 47·8 per cent of the patients were women and the mean age was 71·7 years. Emergency procedures accounted for 29·6 per cent of the cases. The most frequent surgical procedure was right‐sided hemicolectomy, followed by left‐sided resections.

**Table 1:** Patient characteristics and mortality rates according to subgroup categories

	No. of patients	Mortality rate (%)	*P* †
Total no. of patients	129 196	5·8	
Hospital volume			
Resections per hospital 2012–2015*	120·5(91·9)		
Annual resections per hospital	30·1		
Age (years)*	71·7(11·8)		
Age group (years)			< 0·001
≤ 54	11 733	1·2	
55–74	57 948	3·2	
≥ 75	59 515	9·2	
Sex			< 0·001
F	61 800	5·2	
M	67 396	6·3	
Type of resection			< 0·001
Extended (total/subtotal)	15 560	7·6	
Right‐sided	62 278	5·6	
Transverse	4070	6·6	
Left‐sided	43 925	5·0	
Other	3363	8·2	
Emergency procedure	38 181	8·9	
Co‐morbidity score*	100·6(2·4)		
Duration of hospital stay (days)*	19·6(13·5)		

*Values are mean(s.d.). †χ^2^ test.

Overall, the nationwide in‐house mortality for colonic cancer surgery was 5·8 per cent. The mortality rate was higher in the elderly and in men. Extended colonic resection carried a 7·6 per cent risk of in‐hospital death, whereas the mortality rate was less for right‐ and left‐sided colectomies (5·6 and 5·0 per cent respectively) (*Table* 1).

No significant differences were reported in the total number of patients or in mean age during the study period. However, mean LOS decreased over time: 20·4 days in 2012 *versus* 18·8 days in 2015 (*P* < 0·001, data not shown).

### Hospital volumes and mortality

**Figure 2:**
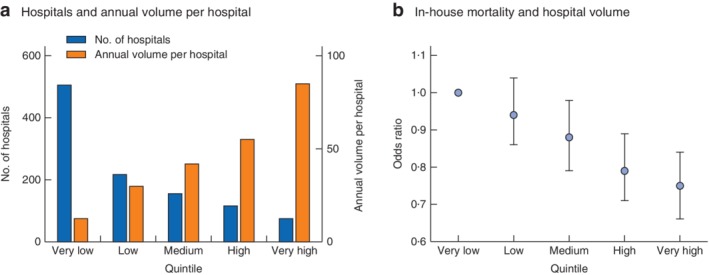
Association of annual hospital volume and in‐house mortality. **a** Number of hospitals and mean annual number of patients treated per hospital according to hospital volume quintiles. **b** Risk‐adjusted odds ratios with 95 per cent confidence intervals for in‐hospital mortality according to hospital volume quintiles

Hospitals were grouped into five case‐load quintiles with approximately the same absolute number of patients (258 392 per quintile, with a maximum absolute difference of 0·3 per cent between volume groups). Some 506 of the 1072 hospitals were in the very low quintile, and 76 hospitals were in the very high‐volume category (*Table* 2 and *Fig*. 2 *a*).

**Table 2:** Hospitals, patient characteristics and mortality rates according to subgroup categories

	Hospital volume quintiles (no. of procedures)					
	Very low (1–97)	Low (98–143)	Medium (144–191)	High (192–259)	Very high (260–1085)	*P* ‡
No. of hospitals	506	217	156	117	76	
Total no. of patients	25 657	25 828	26 091	25 795	25 825	
Volume per hospital, 2012–2015*	50·7(27·5)	119·0(13·3)	167·3(14·4)	220·5(19·7)	339·8(110·7)	< 0·001
Annual volume per hospital*	12·7	29·8	41·8	55·1	85·0	< 0·001
Mortality (%)	1775 (6·9)	1657 (6·4)	1488 (5·7)	1273 (4·9)	1239 (4·8)	< 0·001
Age (years)*	73·0(11·5)	71·9(11·6)	71·7(11·7)	71·0(11·7)	70·7(12·2)	< 0·001§
Women	12 676 (49·4)	12 432 (48·1)	12 418 (47·6)	12 026 (46·6)	12 248 (47·4)	< 0·001
Type of resection†						
Extended (total/subtotal)	2829 (9·7)	2933 (9·5)	3302 (7·3)	3082 (6·4)	3414 (6·0)	< 0·001
Right‐sided	12 649 (6·5)	12 507 (6·4)	12 711 (5·6)	12 161 (4·7)	12 250 (4·9)	< 0·001
Transverse	865 (8·5)	843 (8·2)	826 (5·2)	805 (5·5)	731 (5·3)	0·005
Left‐sided	8522 (6·2)	8823 (5·2)	8563 (5·2)	9181 (4·4)	8836 (4·0)	< 0·001
Emergency procedure†	7874 (9·9)	8080 (9·3)	7609 (8·8)	7352 (8·3)	7266 (8·0)	< 0·001
Co‐morbidity score*	100·7(2·4)	100·7(2·4)	100·6(2·4)	100·5(2·3)	100·5(2·3)	n.a.
Duration of hospital stay (days)*	20·3(12·8)	20·2(13·4)	19·9(13·8)	19·0(13·4)	18·7(13·9)	< 0·001§
Ventilation for > 48 h†	1542 (39·8)	1570 (39·6)	1401 (38·7)	1244 (40·5)	1367 (37·0)	< 0·001
Transfusion ≥ 6 units†	258 (17·1)	281 (16·7)	189 (18·5)	225 (21·8)	259 (18·9)	< 0·001

Values in parentheses are percentages of number of patients in that quintile, unless indicated otherwise; *values are mean(s.d.); †values in parentheses are mortality rates. n.a., Not applicable. ‡χ^2^ test, except §Wilcoxon test for trend.

A mean of 12·7 patients were treated annually in very low‐volume hospitals, whereas very high‐volume hospitals performed 85·0 colonic resections per year. The mean age of the patients varied according to hospital quintile: 73·0 years in the very low‐volume *versus* 70·7 years in the very high‐volume category (*P* < 0·001).

There was a significant inverse association between hospital volume and mortality during hospital stay. Crude in‐house mortality rates ranged from 6·9 per cent (1775 of 25 657 patients) in hospitals in the lowest volume category to 4·8 per cent (1239 of 25 825) in the highest‐volume centres (*P* < 0·001) (*Table* 2).

After stratification for cancer localization, low‐volume hospitals had a significantly higher mortality rate than high‐volume hospitals (*Table* 2).

Mean LOS was similar (20·3 days in very low‐volume hospitals *versus* 18·7 days in very high‐volume hospitals; *P* < 0·001), whereas the percentage of emergency procedures was significantly lower in high‐volume centres (*P* < 0·001).

Univariable analysis documented that sex, age category, co‐morbidity, emergency procedures, prolonged mechanical ventilation and blood transfusion of 6 units or more were significantly associated with in‐house mortality. Mortality increased with fewer patients treated per hospital (*Table* 3).

**Table 3:** Univariable analysis of in‐house mortality

	Crude odds ratio	*P*
Case‐load quintile		
I	1·00 (reference)	
II	0·92 (0·86, 0·99)	0·022
III	0·81 (0·76, 0·88)	< 0·001
IV	0·70 (0·65, 0·75)	< 0·001
V	0·68 (0·63, 0·73)	< 0·001
Sex		
F	1·00 (reference)	
M	1·24 (1·18, 1·30)	< 0·001
Age group (years)		
≤ 54	1·00 (reference)	
55–74	2·79 (2·34, 3·33)	< 0·001
≥ 75	8·70 (7·33, 10·33)	< 0·001
Co‐morbidity score	1·32 (1·31, 1·34)	< 0·001
Emergency procedure		
No	1·00 (reference)	
Yes	2·10 (2·00, 2·20)	< 0·001
Ventilation for ≥ 48 h		
No	1·00 (reference)	
Yes	16·25 (15·36, 17·18)	< 0·001
Transfusion ≥ 6 units		
No	1·00 (reference)	
Yes	3·80 (3·30, 4·40)	< 0·001

Values in parentheses are 95 per cent confidence intervals.

In multivariable regression, accounting for patient clustering within institutions and for the effect of the confounding variables, a highly significant decreasing trend was found for in‐house death following colonic cancer surgery across hospital volume categories. The OR for death was 25 per cent lower in the highest volume centres, 21 per cent in the fourth highest, and 12 per cent lower in the third highest volume category compared with the baseline rate in the lowest volume hospitals. In the multivariable model, the observed decrease in the OR for in‐hospital death between the two lowest volume categories was not significant (*Table* 4). A model with volume category fitted as a linear predictor variable for in‐hospital death performed equally well (OR 0·93, 95 per cent c.i. 0·90 to 0·95; *P* < 0·001). The number of patients was also determined as a continuous variable. This regression model displayed a highly significant linear trend between the number of patients treated and the risk of inpatient death following colonic cancer surgery (OR per individual patient: 0·999, 0·9987 to 0·9994; *P* < 0·001).

**Table 4:** Multivariable logistic regression model for in‐house mortality by volume category including hospital as random effect

	Adjusted odds ratio	*P*
Case‐load quintile		
I	1·00 (reference)	
II	0·94 (0·86, 1·04)	0·242
III	0·88 (0·79, 0·98)	0·015
IV	0·79 (0·71, 0·89)	< 0·001
V	0·75 (0·66, 0·84)	< 0·001
Sex		
F	1·00 (reference)	
M	1·18 (1·12, 1·25)	< 0·001
Age group (years)		
≤ 54	1·00 (reference)	
55–74	2·34 (1·95, 32·81)	< 0·001
≥ 75	6·81 (5·70, 8·13)	< 0·001
Co‐morbidity score	1·18 (1·17, 1·19)	< 0·001
Emergency procedure		
No	1·00 (reference)	
Yes	1·71 (1·62, 1·80)	< 0·001
Ventilation for ≥ 48 h		
No	1·00 (reference)	
Yes	12·08 (11·34, 12·86)	< 0·001
Transfusion ≥ 6 units		
No	1·00 (reference)	
Yes	1·64 (1·37, 1·95)	< 0·001

Values in parentheses are 95 per cent confidence intervals.

## Discussion

In this nationwide population‐based study, overall in‐house mortality following colonic surgery showed a significant correlation with hospital volume. This correlation was documented overall, as well as for different surgical approaches or emergency surgery. In an adjusted model, in‐house mortality was 25 per cent higher in very low‐volume hospitals compared with very high‐volume hospitals.

A major strength of this study is the completeness of the data, as every inpatient treated surgically for colonic cancer in Germany was included. The present findings correlate well with the results of a previous analysis^17^ from the Berlin Cancer Registry that focused on colonic cancer.

Although hospitals are monitored closely by the German statutory assurances' medical services, overreporting or underreporting cannot be excluded completely. In addition, co‐morbidities were included in the regression model using a score validated for German DRG data. This validated score outperforms other commonly used scores^15^. Unfortunately, it was not possible to distinguish between a *de novo* co‐morbid condition, which appeared during the hospital stay, or pre‐existing co‐morbidities.

Finally, no data on 30‐ or 90‐day mortality rates after colonic cancer resection could be provided, and the federal DRG data did not contain information about tumour stage or metastasis.

The results of in‐house or 30‐day mortality are in line with those from previous population‐based studies in this field^9^, ^10^, ^11^. Previous research^12^ has also documented that high‐volume hospitals, surgeons with a specialization in colorectal surgery, and surgeon caseload are associated with better short‐ and long‐term outcomes.

Currently the German Cancer Society certifies oncological care centres and, for colonic cancer, centres have to fulfil several criteria, amongst which is an annual caseload of more than 30 patients with colonic cancer. In 2015, 273 centres were board‐certified and performed a total of 15 627 colonic cancer resections. This accounts for approximately 50 per cent of all colonic and rectal cancer resections in Germany. The remaining patients were treated in uncertified centres. The reported^18^ overall 30‐day mortality rate following colorectal surgery in board‐certified centres was 2·4 per cent. However, these results cannot be compared directly with those found in the present study as emergency cases were included here.

## Disclosure

The authors declare no conflict of interest.

## Supporting information


**Table S1.** DRG and procedure codes
