# Supplementary material for: Nationwide in‐hospital mortality following colonic cancer resection according to hospital volume in Germany
Source: BJS Open. 2019 May 3;3(5):672–7. doi: 10.1002/bjs5.50173 (PMC6773649; doi:10.1002/bjs5.50173)
Supplement: Supplementary file 1 — Table S1. DRG and procedure codes [file BJS5-3-672-s001.docx]

**BJS5_50173**

**Nationwide in-hospital mortality following colonic cancer resection according to hospital volume in Germany**

**J. Diers, J. Wagner, P. Baum, S. Lichthardt, C. Kastner, N. Matthes, S. Löb, H. Matthes, C.-T. Germer and A. Wiegering**

**Table S1 DRG and procedure codes**

|  | **Code** |
| --- | --- |
| **Primary case identifier** | ICD-10-GM (versions 2012-15) |
| Colon cancer | C18 |
| **Retained procedure codes** | OPS versions (for years) |
| Colectomy | 5456 and subclasses (2014-25), 54583/-4 (2013) |
| Subtotal colonic resection | 54559, -a, -b, -c, -d (2013-15), 54582 (2012) |
| Right hemicolectomy | 54554 (2012-15), 54580 (2012) |
| Transverse resection | 545555 (2012-15) |
| Left hemicolectomy | 54556 (2012-15), 54581 (2012) |
| Sigmoid resection | 54557 (2012-15), 54585 (2012) |
| Rectosigmoid resection | 5484 (2012-15) |
| Ileocaecal resection | 54552 (2012-15) |
| Caecal resection | 54553 (2012-15) |
| Resection of >1 segment | 54551 (2012-15) |
| Resection of 1 segment | 54550 (2012-15) |
